# Genome-Wide Analysis Reveals Dynamic Epigenomic Differences in Soybean Response to Low-Phosphorus Stress

**DOI:** 10.3390/ijms21186817

**Published:** 2020-09-17

**Authors:** Shanshan Chu, Xiangqian Zhang, Kaiye Yu, Lingling Lv, Chongyuan Sun, Xiaoqian Liu, Jinyu Zhang, Yongqing Jiao, Dan Zhang

**Affiliations:** 1 Collaborative Innovation Center of Henan Grain Crops, College of Agronomy, Henan Agricultural University, Zhengzhou 450046, China; chushan3@163.com (S.C.); 15333717403@163.com (X.Z.); 17638110601@163.com (K.Y.); 13525606625@163.com (L.L.); 13183019220@163.com (C.S.); liuxq2019@126.com (X.L.); jiaoyongqing@henau.edu.cn (Y.J.); 2 Collaborative Innovation Center of Modern Biological Breeding, Henan Institute of Science and Technology, Xinxiang 453003, China; zjy891@126.com

**Keywords:** DNA methylation, epigenetics, low-phosphorus stress, gene expression, small RNA, soybean

## Abstract

Low-phosphorus (low-P) stress has a significant limiting effect on crop yield and quality. Although the molecular mechanisms of the transcriptional level responsible for the low-P stress response have been studied in detail, the underlying epigenetic mechanisms in gene regulation remain largely unknown. In this study, we evaluated the changes in DNA methylation, gene expression and small interfering RNAs (siRNAs) abundance genome-wide in response to low-P stress in two representative soybean genotypes with different P-efficiencies. The DNA methylation levels were slightly higher under low-P stress in both genotypes. Integrative methylation and transcription analysis suggested a complex regulatory relationship between DNA methylation and gene expression that may be associated with the type, region, and extent of methylation. Association analysis of low-P-induced differential methylation and gene expression showed that transcriptional alterations of a small part of genes were associated with methylation changes. Dynamic methylation alterations in transposable element (TE) regions in the CHH methylation context correspond with changes in the amount of siRNA under low-P conditions, indicating an important role of siRNAs in modulating TE activity by guiding CHH methylation in TE regions. Together, these results could help to elucidate the epigenetic regulation mechanisms governing the responses of plants to abiotic stresses.

## 1. Introduction

The processes of plant growth and development are generally subject to various environmental stresses, including biotic and abiotic stress. During evolution, plants gradually evolved elaborate sensory and adaptive mechanisms, including changes at the physiological and biochemical levels, to better adapt to adverse environmental conditions [1]. Recent studies have identified a large number of genes encoding transcriptional factor regulation of gene transcription, enzymes involved in stress signal transduction, and functional proteins that change downstream cell status participating in plant stress responses [2]. Furthermore, epigenetic regulation factors have been suggested to play an important role in the transcriptional and posttranscriptional control of these genes [3]. DNA methylation is one of the most well-studied epigenetic markers [4,5] that modulate gene expression in response to both biotic and abiotic stresses [6,7].

DNA methylation exists in three sequence contexts, including CG, CHG, and CHH (where H = A, C, or T), by activating different DNA methyltransferase enzymes and the RNA-directed DNA methylation (RdDM) pathway in plants [8,9,10]. DNA methylation in the symmetric CG and CHG contexts is copied during DNA replication and established by conserved methyltransferase1 (MET1) and the plant-specific DNA methyltransferase chromomethylase 3 (CMT3), respectively [5,11,12,13]. DNA methylation in the nonsymmetrical CHH context is generated de novo after DNA replication and established by the small (typically 24 nucleotides) interfering RNA-directed DNA methylation (RdDM) pathway [3]. The extent of genomic DNA methylation is maintained not only by the DNA methylation process but also by the DNA demethylation process catalyzed by several DNA demethylases, such as the DEMETER family [14,15].

Previous investigations on the alterations of DNA methylation coping with stresses have utilized low-resolution and nonquantitative methods [16,17,18,19]. Nevertheless, the emergence of high-throughput genomic sequencing technology enables single-base-resolution analysis of DNA methylation in the genome-wide range [20,21], thereby enabling global assessment of the pattern changes of DNA methylation responding to various environmental cues. Numerous studies have shown that environmental stress on plants could significantly induce changes in methylation levels in genes companied with changes in transcriptional abundance. In poplar (*Populus trichocarpa*), drought stress induced widespread alterations in DNA methylation [22]. Moreover, the extent of changes in genomic DNA methylation has affected abundant drought-related transcriptional changes [23]. Additionally, environmental stresses also changed epigenetic variations in transposable element (TE), indicating that TEs are involved in the plant stress response with epigenetic alterations [7]. For instance, a Tam3 transposon methylation alteration at CHH sites in snapdragon (*Antirrhinum majus*) was detected in response to low-temperature stress [24]. In addition, the Mutator element MuDR was demethylated along with the increased expression level of the mudrA transposase gene in maize responding to low nitrogen ion stress [25]. These investigations revealed that environmental stresses could generate significant effects on DNA methylation alterations and TE mobilization.

In natural and agricultural ecosystems, one of the most common abiotic stresses is low phosphorus (P) availability [26,27], which restricts crop productivity in more than 70% of globally available arable land [28]. To overcome the issues of low availability of inorganic P in the soil [29], applying a large amount of P fertilizer has become the main strategy to maintain crop yield. However, excessive P application not only increases the input-output ratio but also causes the accumulation of harmful elements in the soil and environmental pollution. Even more worrying is that phosphorus resources are not renewable, and the world’s available phosphate mines will be depleted in the next 50–80 years at current mining rates [27]. Therefore, a better understanding of the molecular mechanism involved in P homeostasis to improve the absorption and utilization efficiency of P in crops is a sustainable way to improve global food security.

Plants have evolved a range of sophisticated responses aimed at coping with low P availability [2,30]. In low-P stress, the primary root is responsible for sensing local low-P signals [31], and transcription factors, such as PHR1 and PHL1, are responsible for modulating long-distance phosphate signaling in *Arabidopsis thaliana* [32]. Both *PT* genes encoding high-affinity phosphate transporters and *ACP* genes encoding acid phosphatase could increase phosphate uptake by improving expression levels. In addition, SPX-domain-containing proteins, such as SPX and PHO1, have been reported to mediate the regulation of phosphorus homeostasis [30,33,34,35,36,37,38]. 

Despite the considerable advances in understanding transcriptional and posttranscriptional mechanisms of plant responses to low P availability, several epigenetic mechanisms regulating gene expression coping with this stress have only been assessed by a limited number of studies that are confined to few model organisms [39,40,41]. For example, in *Arabidopsis*, global DNA methylation occurs with extensive remodeling under low-P stress, which is associated with changes in P starvation response gene expression. This study revealed that dynamic methylation changes play pivotal roles in response to P starvation [40]. Soybean is the main source of human edible oils and vegetable proteins. Compared with nonlegumes, such as rice or corn, soybean requires more P because of the higher P content in soybean seeds [42]. P deficiency in soybeans not only affects the growth and development of plants and increases the loss of flower, as well as pods, but also affects the formation of nodules, thereby reducing nitrogen fixation efficiency and ultimately affecting its quality and yield [43,44,45]. Therefore, soil P deficiency has become an important factor limiting the development of soybean production [46]. Accordingly, it is particularly important to understand the epigenetic regulation mechanism of the low-P stress response in soybean. 

In our study, we constructed DNA methylation maps with single-base resolution and genome-wide coverage in two representative soybean genotypes with different P efficiencies, ‘Nannong 94156′ and ‘Bogao’ (a tolerant genotype and a sensitive genotype to low-P stress, respectively) under low-P (LP, -P, 5 μM) and high-P (HP, +P, control, 500 μM) conditions, respectively. This investigation was designed to answer two main questions: (i) the genomic landscape and changes in the soybean methylome associated with low-P stress (ii) and the relationship between methylome alterations and P-efficiency-associated gene expression alterations.

## 2. Results

### 2.1. Genome-Wide DNA Methylation Patterns in Response to Low-P Stress

To understand the genomic DNA methylation features and patterns at a single nucleotide in response to P availability, we examined the global DNA methylation levels in root tissues of the representative low-P-tolerant ‘NN’ and low-P-sensitive ‘BG’ cultivars by single-base resolution whole-genome bisulfite sequencing [5]. In total, our bisulfite sequencing yielded 366193864-522038932 raw reads for each of the four DNA library samples (Table 1). After removal of adapter contaminants, low-quality reads and reads containing Ns, 360336480-515810512 clean reads were collected (Table 1), of which approximately 88% were mapped into the soybean genome. Moreover, approximately 67% of cytosines were covered by more than one uniquely mapped read in the soybean genome. The sequencing data were ready for further analysis, while the sequencing depth reached 50×, and the detected cytosine number reached saturation (Figure S1). Bisulfite conversion efficiency ranged from 99.62% to 99.66% per sample, as determined using the nonmethylated λ phage genome (Table 1).

**Figure 1:**
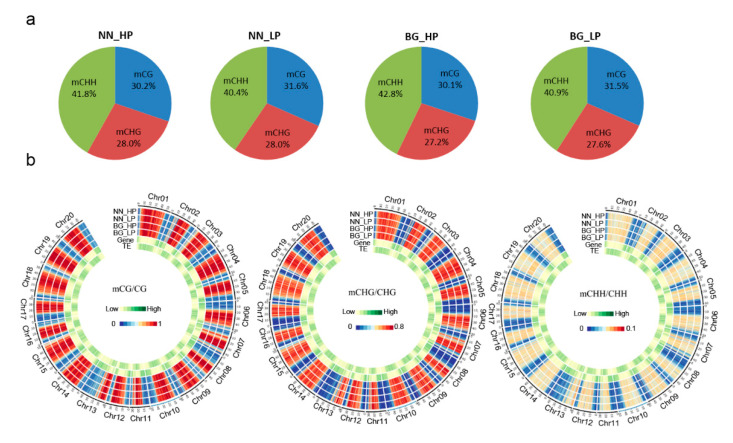
DNA methylome features in soybean. (**a**) Relative proportions of mCs in three sequence contexts (CG, CHG, and CHH); (**b**) A circos plot of gene and transposon density and mCG, mCHG, and mCHH location in soybean. NN_HP represents ‘Nan-nong94-156′ under control conditions; NN_LP represents ‘Nan-nong94-156′ under low-P conditions; BG_HP represents ‘Bogao’ under control conditions, and BG_LP represents ‘Bogao’ under low-P stress.

The NN_HP (‘Nan-nong94-156′_high P) genome presented 66.50% mCG (mCG/CG), 43.63% mCHG (mCHG/CHG), and 3.68% mCHH (mCHH/CHH), which showed the percentage of methylation levels in the soybean genome. Correspondingly, BG_HP (‘Bogao’_high P) presented 67.48%, 44.00%, and 3.84% in CG, CHG, and CHH contexts, respectively (Table S1 and Figure S2). We found that ‘NN’ and ‘BG’ exhibit a similar pattern in response to P availability (LP vs. HP) and that all three sequence contexts of DNA methylation levels were slightly higher after low-P stress. While investigating the distributions of mCs in three sequence contexts, we observed that methylcytosine was most common at the CHH sites (40.4–42.8%) and occurred less frequently in CG and CHG sequences (30.1–31.6% and 27.2–28.0%, respectively). A slight increase in CG and CHG methylation proportions and a decrease in CHH methylation was found in both ‘NN’ and ‘BG’ under low-P stress (Figure 1a). Global DNA methylation profiles demonstrated that a high degree of methylation occurred in transposable element (TE)-rich regions, while the gene-rich regions exhibited relatively reduced methylation in the soybean genome (Figure 1b). This result was similar to previous findings in soybean [47], *Arabidopsis* [48], and rice [49], suggesting that DNA methylation in transposon silencing might be conserved in plants.

### 2.2. DNA Methylation Patterns in Gene and TE Regions

**Figure 2:**
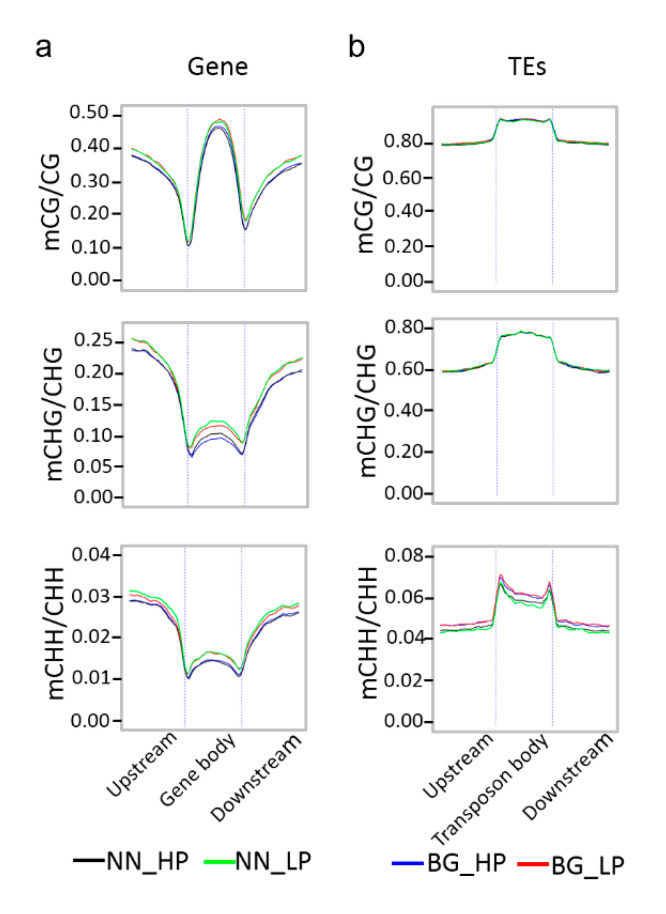
Genomic DNA methylation profiles in soybean. (**a**) DNA methylation patterns across genes; (**b**) DNA methylation patterns across TEs; the vertical dashed line represents the boundaries of the gene body or transposon (TE) body.

While inspecting the distribution of CG, CHG, and CHH methylations in gene and TE regions, we observed that CG methylation occurred preferentially in the gene body regions relative to the flanking regions, similar to previous reports in other plants [47,48,50,51], whereas the extents of CHG and CHH methylation were low in gene body regions and relatively higher in flanking regions (Figure 2a). The DNA methylation extents of the CG, CHG, and CHH contexts were notably low near transcriptional start sites and transcriptional end sites but increased gradually with increasing distance from these sites (Figure 2a). In contrast to gene body regions, the TEs were highly methylated in all CG, CHG, and CHH sequence contexts (Figure 2b). Furthermore, we noted that most of the methylated TEs belonged to class I (retro-transposons), especially for LTR/Gypsy and LTR/Copia, consistent with their abundances in the soybean genome. Among class II (DNA transposons), the TE-type DNA/MuDR was more frequently methylated than others (Figure S3a).

Unsurprisingly, the low-P treatment clearly exhibited CG, CHG, and CHH hypermethylation in both the gene body and flanking regions compared with the high phosphorus treatment (Figure 2a). In TE regions, striking differences of methylation levels were observed in the CHH methylation context, while no significant differences were observed in CG and CHG methylation contexts between different P levels treatments. The NN_LP exhibited a lower CHH methylation level in TE regions than in the NN_HP (Figure 2b). Consistent with the similar methylation extent of TEs in both phosphorus treatments, the average methylation extents of both class I and II TEs are largely the same in CG and CHG contexts between different phosphorus treatments. The TEs within class I exhibited a higher methylation extent than those within class II in CG and CHG contexts, whereas the CHH methylation extent of the class II TEs appeared to be significantly higher compared with the class I TEs (Figure S3b). Interestingly, in NN_LP, the reduced CHH methylation extent of TEs may be due to the lower average methylation level of class I TE (Figure S3b).

### 2.3. Association Analyses of DNA Methylation Status and Gene Expression 

**Figure 3:**
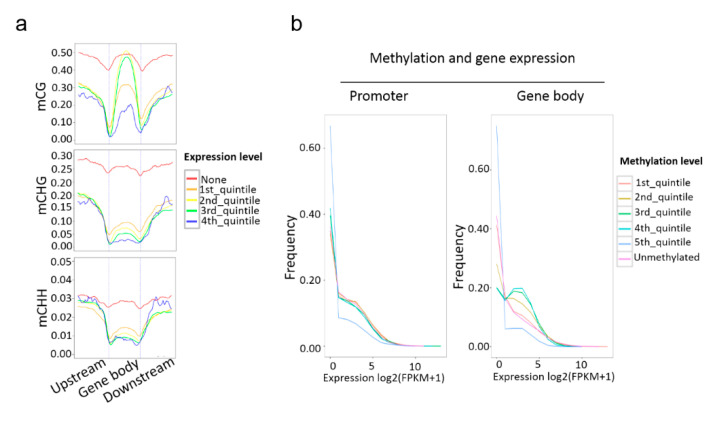
Relationship between DNA methylation and gene expression. (**a**) Distributions of methylation levels within gene bodies partitioned by different expression levels: 1st_quintile is the lowest and 4th_quintile is the highest; genes with FPKM value < 0.1 were considered non-expressed (none); (**b**) expression profiles of methylated genes compared with unmethylated genes. Methylated genes were further divided into quintiles based on promoter and gene body region methylation levels: 1st_quintile is the lowest and 5th_quintile is the highest.

DNA methylation controls genes in numerous biological processes. To unveil how the promoter and gene body methylation functions in gene expression, transcriptome profiles of low-P-treated ‘NN’ and ‘BG’ were generated. These materials are identical to the ones for methylome analysis. Genes were separated into two parts, the non-expressed (none) genes (FPKM value < 0.1) and the expressed genes. Based on the expression level, the expressed genes were further divided into four groups in ascending order. Non-expressed genes maintained relatively higher methylation levels as expected in all three sequence contexts (Figure 3a). Correspondingly, expressed genes with the highest expression levels had the lowest CG and CHG methylation levels in gene body and flanking regions, and the lowest CHH methylation levels in gene body regions (Figure 3a). The moderately expressed genes had moderate CG, CHG, and CHH methylation levels in gene body regions. However, the expressed genes with low or moderate expression levels showed lower CHH methylation levels than the highly expressed genes in promoter and downstream 2-kb regions (Figure 3a).

For further study of the connection between gene methylation and expression, genes were categorized as methylated or unmethylated according to the methylation level. Methylated genes were ranked based on the promoter or gene body methylation levels. Then, the genes were sorted into five groups accordingly (Figure 3b). The first 20% was the genes involved with the lowest methylation level, while the fifth group was the highest. The Figure 3b shows that there is an inverse correlation between promoter methylation and gene expression, since higher levels of promoter methylation show lower expression levels. Consistent with these results, the gene body with the highest methylation levels showed the lowest expression levels, but there was no significant difference among the other methylation-level groups. In addition, we performed a Spearman correlation analysis between methylation and gene expression levels. As shown in Figure S4, the overall correlation rho was low regardless of the methylation types. However, the rho can reach −0.22 in downstream 2-kb regions for CG methylation. This result suggested that methylation levels in a small fraction of downstream 2-kb regions have relatively higher correlation with their expression levels.

### 2.4. Identification of DMR and DMR-Associated Genes in Response to Low-P Stress

To explore the possible influence of low-P stress on methylation, we identified the differentially methylated regions (DMRs) between NN_LP vs NN_HP and BG_LP vs BG_HP (‘NN’ low-P versus high-P and ‘BG’ low-P versus high-P) by comparing fractional methylation levels of 10 kb windows throughout the genome (FDR < 0.05). In response to low-P stress, we found more low-P-inducible hyper-DMRs (i.e., higher methylation in low-P-treated accessions) in all contexts in both ‘NN’ and ‘BG’ and CHH-DMRs were most abundant among all methylation contexts (Figure S5). The cluster analysis also revealed a widespread methylome change under low-P stress (Figure S6). Among these CG DMRs, approximately 46–49% of DMRs were located in TE regions, 31–34% of DMRs were located in the gene body region, while only 10% of DMRs were located in 2-kb upstream and downstream regions of the gene, respectively. Similarly, most CHG DMRs (approximately 60%) were found in TE regions, 17–18% of DMRs were found in the gene body region, and only 11–12% of DMRs were found in upstream and downstream regions, respectively. For the CHH DMRs, most of them (approximately 41–45%) were also located in TE regions, 21–27% were located in the upstream or downstream regions, while only 11% of DMRs were located in the gene body region (Figure S7). Briefly, most DMRs in all three contexts were found in the TE regions. These analyses provided a clear landscape of genomic methylation differentiation in BG_LP vs BG_HP and NN_LP vs NN_HP.

**Figure 4:**
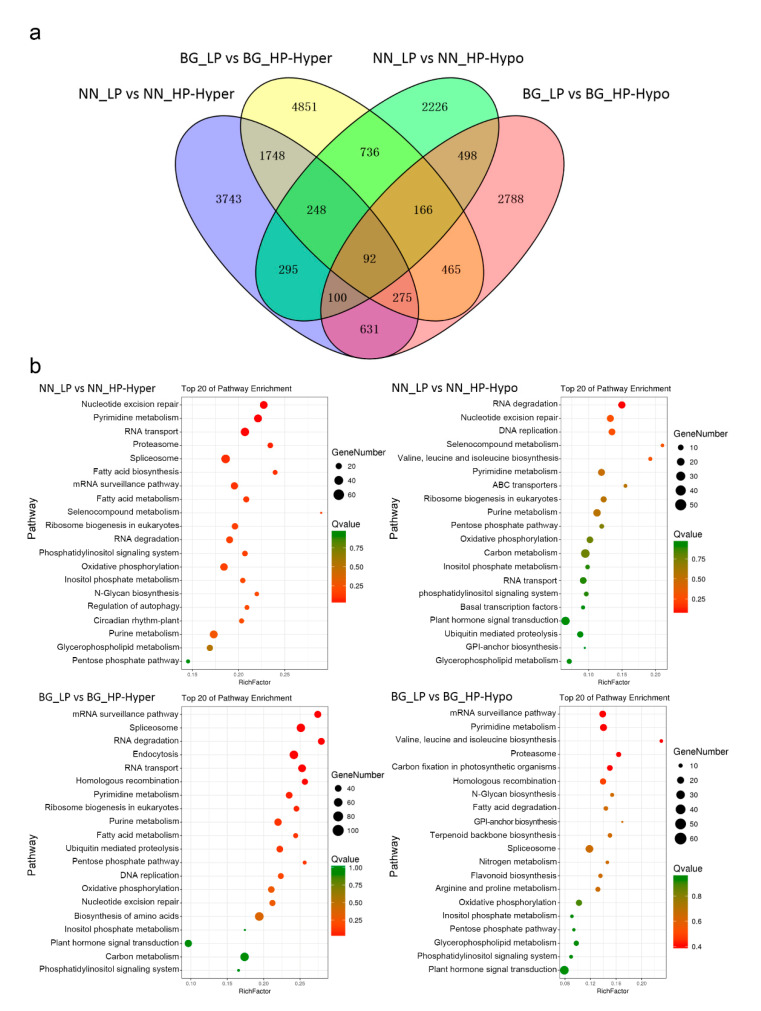
Differential methylome analysis under low-P stress. (**a**) Venn diagram of hyper/hypomethylated genes among ‘Nan-nong94-156′ and ‘Bogao’ under low-P stress; (**b**) KEGG pathway enrichment of hypermethylated and hypomethylated genes in two cultivars under low-P conditions. The size of the circle represents gene numbers, and the color represents the q-value. NN_LP vs. NN_HP, ‘NN’ low-P versus high-P; BG_LP vs BG_HP, ‘BG’ low-P versus high-P.

**Figure 5:**
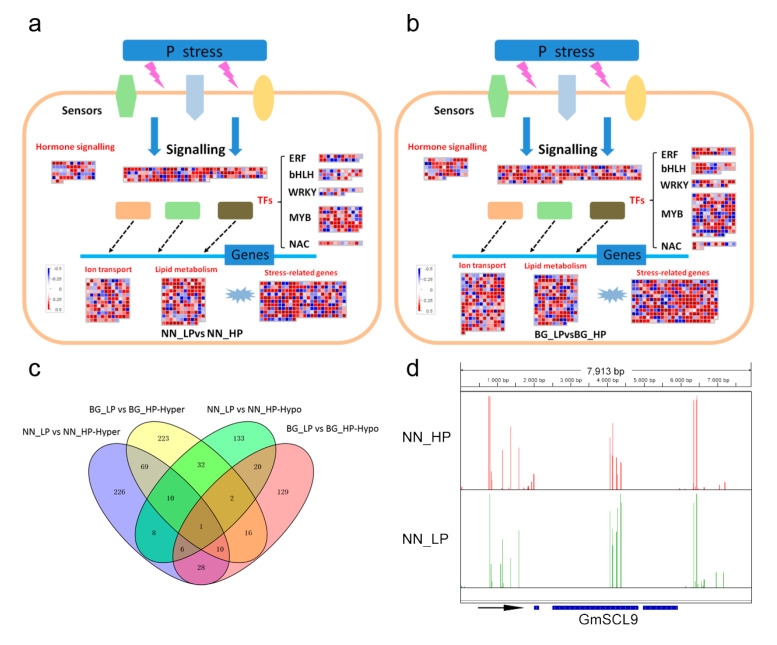
Assignment of differentially methylated genes among ‘Nan-nong94-156′ (**a**) and ‘Bogao’ (**b**) under low-P stress in Mapman bins. The red and blue squares indicate the hyper- and hypomethylated genes, respectively. Other different shaped graphics with different colors refer to some sensors and transcription factors (TFs) responding to low-P stress. (**c**) Venn map of differentially methylated transcriptional factors; (**d**) IGV software depicts the hypermethylation of GmSCL9 gene body region induced by low-P stress in ‘Nan-nong94-156′. NN_LP vs NN_HP, ‘NN’ low-P versus high-P; BG_LP vs BG_HP, ‘BG’ low-P versus high-P.

Some overlap between DMRs and genes was detected based on the association between the DMR positions with protein-coding genes and 2 kb upstream and downstream regions. In total, 7132 hypermethylated and 4361 hypomethylated genes were identified in NN_LP vs NN_HP; similarly, 8581 hypermethylated genes and 5015 hypomethylated genes were identified in BG_LP vs BG_HP (Table S2). ‘NN’ and ‘BG’ shared 2363 hypermethylated genes and 856 hypomethylated genes under low-P stress (Figure 4a). To obtain a deeper understanding of the potential biological functions of DMR genes, a Gene Ontology (GO) category analysis was performed. We found that these DMR genes were primarily associated with nucleic acid, phosphorus, and nitrogen compound metabolic processes, stressing response, macromolecular complex, and ion binding, regardless of whether they are hypermethylated genes or hypomethylated genes (Figure S8). Furthermore, we identified pathways affected by low-P treatment in both genotypes using KEGG (Kyoto Encyclopedia of Genes and Genomes) pathway enrichment analysis. As shown in Figure 4b, hypermethylated genes in NN_LP vs NN_HP show abundant enrichment in pathways related to such processes as nucleotide excision repair, oxidative phosphorylation, spliceosome, and RNA transport. The last two pathways are also enriched in BG_LP vs BG_HP. Among hypomethylated genes in NN_LP vs NN_HP, pathways of RNA degradation, carbon metabolism, and plant hormone signal transduction were enriched. In addition to the last pathway, hypomethylated genes in BG_LP vs BG_HP are also assigned in pathways related to mRNA surveillance, pyrimidine metabolism, and spliceosome. Figure 5a,b show the detailed distribution of differentially methylated genes (DMGs) in pathways by Map-Man. Plentiful DMGs were involved in pathways of ‘signaling’ and hormone signaling. Moreover, low-P-related transcription factors, including *ERF*, *bHLH*, *WRKY*, *NAC,* and *MYB* members, involved differences in methylation. In addition, the methylation levels of genes played roles in ion transport, lipid metabolism, and stress response were also disrupted (Figure 5a,b).

While investigating the change in methylation of transcription factors (TFs) under low-P stress, 570 TFs showed alterations in methylation in NN_LP vs NN_HP. The TFs consisted of 358 hypermethylated genes and 212 hypomethylated genes. A total of 363 and 212 of 575 TFs showed hyper- or hypomethylation in BG_LP vs BG_HP, respectively (Table S3). The two cultivars shared 90 common hypermethylated TFs and 29 hypomethylated TFs under low-P stress (Figure 5c). Previous studies reported the different tolerance to P of ‘NN’ and ‘BG’ indicated that ‘NN’ is a low-P-tolerant accession and ‘BG’ is a low-P-sensitive accession. To explore the potential differentially methylated TFs yielding the difference of P tolerance in ‘NN’ and ‘BG,’ we analyzed the noncommon methylation alterations among NN_LP vs NN_HP and BG_LP vs BG_HP. Interestingly, a *GRAS* transcription factor family member, *SCARECROW-LIKE* (*SCL9*), presented remarkable gene body hypermethylation in NN_LP vs NN_HP (Figure 5d) but was unaltered in ‘BG’ under low-P conditions (Figure S9). Moreover, the expression analysis showed that *GmSCL9* mRNA abundance was repressed in NN_LP vs NN_HP but slightly induced in BG_LP vs BG_HP (Table S4). *Arabidopsis SCL9* is homologous to *gibberellin-insensitive* (*AtGAI*) and repressor of *ga1-3* (*AtRGA*), which act as negative regulators of GA signal transduction, and the inactivation of which largely modulate growth-promoting effect on primary roots [52]. Correspondingly, *GmSCL9* was downregulated in ‘NN’ (low-P-tolerant) and non-significantly upregulated in ‘BG’ (low-P- sensitive) under low-P stress (Table S4). These results indicated that the difference in low-P-tolerance between ‘NN’ and ‘BG’ might be due to the differential expression of *GmSCL9* in ‘NN’ and ‘BG.’ Figure S10 illustrates the six additional typical hypomethylation regions, including *WRKY*, which encode a homolog of *AtWRKY6* (AT1G62300) in *Arabidopsis* and modulates *Phosphate1 (Pho1*) expression in response to low-P stress, *ERF*, *bHLH*, *MYB*, *ARF,* and *NAC* (Figure S10). 

### 2.5. Abundant TE Genes Are Hypomethylated in Response to Low-P Stress

**Figure 6:**
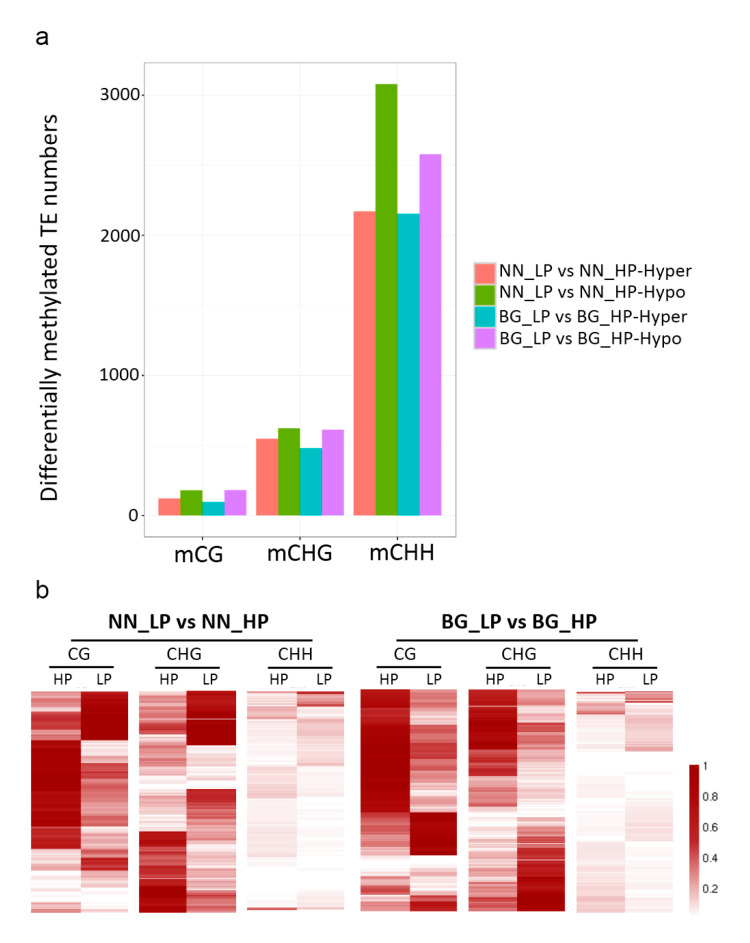
Differentially methylated TEs. (**a**) Numbers of differentially methylated TEs in ‘Nan-nong94-156′ or ‘Bogao’ under low-P stress; (**b**) heat maps of differentially methylated TEs. NN_LP vs NN_HP, ‘NN’ low-P versus high-P; BG_LP vs BG_HP, ‘BG’ low-P versus high-P.

Transposable elements (TEs) are mobile DNA elements within the genome and their mobilization and silencing were reported to be associated with DNA methylation disruption [7,24,53]. The data showed that a higher number of TEs were associated with hypo-DMRs in both genotypes under low-P stress (Figure 6a). For NN_LP vs NN_HP, 123, 548, and 2172 hypermethylated and 179, 623, and 3079 hypomethylated TEs in mCG, mCHG, and mCHH sites were identified, respectively. For BG_LP vs BG_HP, 96, 480, and 2153 TEs were hypermethylated in mCG, mCHG, and mCHH contexts, respectively, whereas 182, 612, and 2578 TEs were hypomethylated (Figure 6a). As shown in the heat maps in Figure 6b, the methylation changes were further exhibited in differentially methylated TEs in each methylation context among NN_LP vs NN_HP and BG_LP vs BG_HP. The results suggested that abundant differentially methylated TEs showed demethylation at each methylation sequence context among NN_LP vs NN_HP and BG_LP vs BG_HP. Moreover, the NN_LP vs NN_HP combination presented much more hypomethylated TEs in the CHH context in contrast with BG_LP vs BG_HP. It was reported previously that methylation levels within TEs might dynamically regulate the expression of transposon genes and the near genes in the process of coping with stress [54]. Additionally, the demethylation effect on TEs in this study may be related to the regulation of transposons and genes involved in the response to low-P stress.

### 2.6. Conjoint Analysis of Methylome and Transcriptome Alterations in Low-P Stress

**Figure 7:**
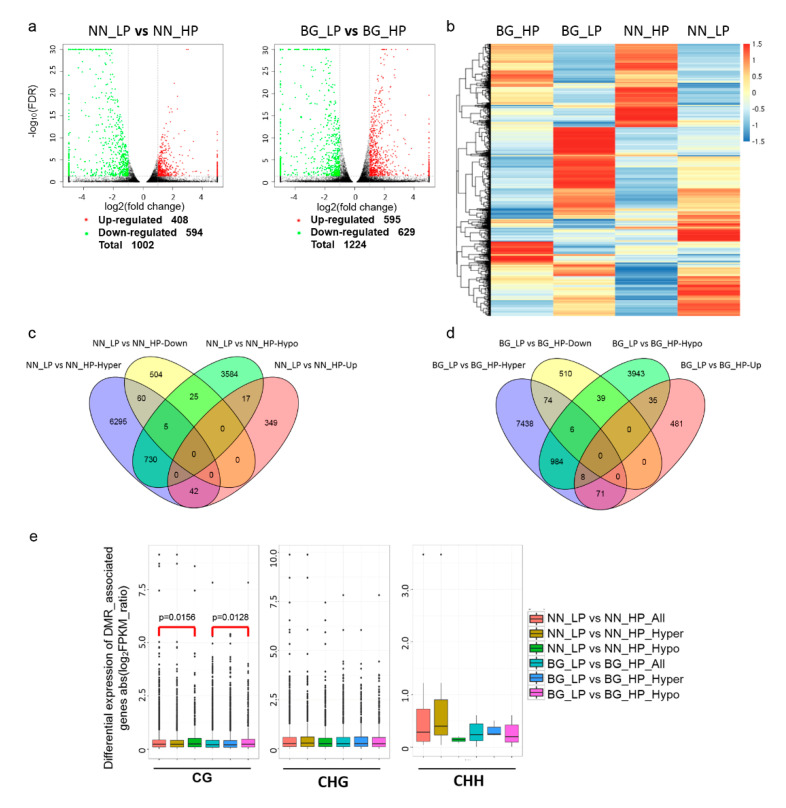
The effect of methylation changes on transcriptional alterations. (**a**) Differentially expressed genes (DEGs) in ‘Nan-nong94-156′ or ‘Bogao’ in response to low-P stress. Each dot represents one gene. The red dots represent upregulated genes and the green dots represent downregulated genes. The black dots represent genes without differential expression. The X-axis is the log2 value of fold change and the Y-axis is the log10 value of false discovery rate (FDR); (**b**) heat maps of DEGs. NN_HP, ‘Nan-nong94-156′ under control conditions; NN_LP, ‘Nan-nong94-156′ under low-P conditions; BG_HP, ‘Bogao’ under control conditions; BG_LP, ‘Bogao’ under low-P conditions. Venn diagram of DMGs (differentially methylated genes) and DEGs in NN_LP vs NN_HP (**c**) and BG_LP vs BG_HP (**d**); (**e**) differential expression levels of all genes (red box), hypermethylated genes (green box), and hypomethylated genes (blue box) among NN_LP vs NN_HP and BG_LP vs BG_HP in three sequence contexts (CG, CHG, and CHH) are displayed as boxplots (boxes represent the quartiles; Wilcoxon P values are reported). NN_LP vs NN_HP, ‘NN’ low-P versus high-P; BG_LP vs BG_HP, ‘BG’ low-P versus high-P.

To explore the gene expression alterations accompanied by widespread methylation changes in response to low-P stress, RNA-seq analysis was performed with the same accessions grown either under HP or LP conditions. In total, 1002 and 1224 genes were differentially expressed in ‘NN’ and ‘BG,’ respectively (Table S4). In brief, 408 genes were upregulated and 594 genes were downregulated in NN_LP vs NN_HP, while 595 upregulated genes and 629 downregulated genes were present in BG_LP vs BG_HP (Figure 7a). These results suggested that more differentially expressed genes (DEGs) were identified in ‘BG’ than in ‘NN’ and that there was a tendency for more downregulated genes in both ‘NN’ and ‘BG’ upon low-P treatment. The hierarchical clustering analysis of genome-wide transcriptional alterations showed distinct differences in the way of the tolerant and sensitive accessions responding to the low-P stress (Figure 7b). To investigate the effect of methylation changes on transcriptional alterations, we identified low-P-induced DMGs associated with DEGs. Altogether, 65 hyper-DMGs overlapped with downregulated DEGs, and 17 hypo-DMGs overlapped with upregulated DEGs in NN_LP vs NN_HP. Nevertheless, 42 upregulated DEGs and 30 downregulated DEGs overlapped with hyper-DMGs and hypo-DMGs, respectively (Figure 7c). Similarly, in BG_LP vs BG_HP, 80 DEGs were downregulated with hypermethylation, and 43 DEGs were upregulated with hypomethylation. However, 79 upregulated DEGs and 45 downregulated DEGs overlapped with hyper-DMGs and hypo-DMGs, respectively (Figure 7d).

A list of the low-P-induced and methylation-changed genes and their gene IDs, methylation levels, the corresponding associated expression patterns, and functional annotation is shown in Table S5. Furthermore, we compared the expression levels of all genes with hyper-DMGs or hypo-DMGs. As shown in Figure 7e, in both ‘NN’ and ‘BG’ under low-P stress, hypo-DMR genes showed significantly higher expression levels compared with all genes in the mCG context. In the mCHG context, both hyper-DMGs and hypo-DMR genes did not present significant differences in expression levels compared to all genes. Additionally, in the mCHH context, although the presence of slightly higher expression levels of hyper-DMR genes and the slightly lower expression levels of hypo-DMR genes were detected compared with all genes, there was no statistically significant difference with *p* > 0.05 (Wilcoxon test) between hypomethylated or hypermethylated genes and all genes.

This finding indicates that there was no association between most of the transcript abundance varieties and methylation alterations. Altogether, these data indicate that DNA methylation is partially involved in the transcriptional changes of these genes. A portion of the differentially expressed genes is more a consequence of methylation-dependent alterations in transcriptional networks than a direct target of DNA methylation.

### 2.7. Association Analyses of DNA Methylation and Small RNA Expression

As increasing evidence has indicated that de novo DNA methylation is mediated by RNA-directed DNA methylation (RdDM) pathways, which are guided by small RNAs, we investigated the relationship between small RNA expression and DNA methylation [8,55,56]. The small RNA expression profiles of the same materials as methylome analysis were achieved by high-throughput deep sequencing. As RdDM is guided mainly by 24-nucleotide (nt) small interfering RNAs (siRNAs) and the 24-nt class was the most abundant group of small RNAs in soybean roots based on their length distribution (Figure S11), we focused on the 24-nt siRNA covered regions for subsequent investigations [57].

**Figure 8:**
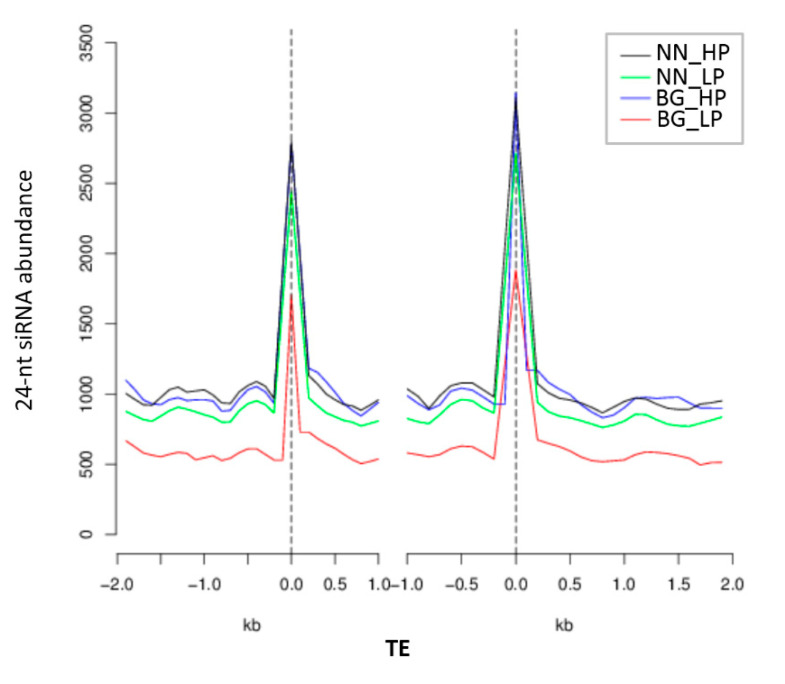
Number distribution of siRNAs in the TE and flanking 2-kb regions. NN_HP represents ‘Nan-nong94-156′ under control conditions; NN_LP represents ‘Nan-nong94-156′ under low-P conditions; BG_HP represents ‘Bogao’ under control conditions, and BG_LP represents ‘Bogao’ under low-P conditions.

We compared the methylation levels between 24-nt siRNA covered regions and the regions without siRNA coverage in each methylation context. The results showed that the methylation level in all three methylation contexts in siRNA regions was significantly increased compared with the regions without siRNA (Figure S12). In addition, the 24-nt siRNA abundance in TE regions rises to a peak near transcriptional start sites and transcriptional end sites and decreases sharply when departing from these sites (Figure 8). In particular, the 24-nt siRNA abundance through all the TE regions in both ‘BG’ and ‘NN’ in LP conditions was substantially lower than that in HP conditions, which was consistent with the CHH methylation pattern in the same region (Figure 2b and Figure 8). These results indicated that 24-nt siRNAs could be responsible for the reduction of the DNA methylation level, especially for the CHH sequence contexts (Figure 2b and Figure 8).

## 3. Discussion

DNA methylation has become one of the most heavily researched topics in plant functional genomics because of its important role in modulating plant plasticity in response to various stresses [47]. Low-P stress is one of the most important abiotic stresses in soybean. Using the whole-genome bisulfite sequencing approach, we evaluated changes in methylation genome-wide when suffering low-P stress in two soybean varieties with different levels of low-P tolerance. Our results revealed that DNA methylation levels were slightly higher under low-P stress and low-P-induced methylome changes partially related to the changes in gene expression and siRNA abundance. The available data sets of our study could be applied to select potent epigenetic regions as probable targets for genetic manipulation strategies for crop improvement to engineer tolerance against abiotic stresses.

Whole-genome bisulfite sequencing (WGBS) in single-base resolution provided an overall view of methylation patterns of the two soybean accessions, presenting an average DNA methylation level of 67.54% mCG (mCG/CG), 44.57% mCHG (mCHG/CHG), and 3.79% mCHH (mCHH/CHH), respectively. Clearly, soybean methylation levels are moderate among diverse reported species. Considering the moderate genome size (978Mb) of soybean, our results provide evidence to confirm the positive correlation between methylation levels and genome sizes [58,59]. It was reported previously that the representative hypermethylation regions were centromeres and peri-centromeric areas. Moreover, the negative correlation between methylation levels and gene number was revealed by several previous studies [21,59,60]. Consistently, our results also demonstrated the positive correlation of CG, CHG, and CHH methylation levels with TE density and the negative correlation with gene number, which suggests the maintenance of genome stability as a primary function of DNA methylation.

DNA methylation has been reported to repress gene expression [61]. However, in recent years, numerous genomic methylation investigations have shown that the correlation between DNA methylation and transcription is slightly different than initially recognized. For example, the recent rice methylome analysis showed that gene body methylation usually presented a positive correlation with gene expression [62]. An investigation in *Arabidopsis* revealed that DNA methylation was only marginally responsible for gene expression [63]. Additionally, a more recent study in apple indicated that there is no apparent relationship between promoter methylation and gene expression [64]. However, in our study, the results showed that non-expressed genes possessed relatively high methylation levels in each methylation sequence context, and the CG and CHG methylation levels exhibited an inverse correlation with expression throughout broad regions of the gene. Surprisingly, although the CHH methylation level was negatively correlated with expression in the gene body, the CHH methylation level of highly expressed genes was higher than expressed genes with low or moderate expression levels in promoter and downstream regions (Figure 3a).

Furthermore, genes with the highest methylation levels showed the lowest expression levels in both promoter and gene body regions, while no significant difference was found among the different methylation-level quintiles. In short, all the results indicated a more complex regulatory correlation between DNA methylation and gene expression which appears to depend on the type, region, and extent of methylation, as well as species.

Low-P stress is one of the most important abiotic stresses, while related DNA methylation pattern studies only focused on a few model plants, such as *Arabidopsis* and rice [39,40,41]. Our study is the first to report the DNA methylation alterations in response to low-P stress in legume model plant soybean. In *Arabidopsis*, P starvation could lead to a series of changes in root architecture, including the great increase in density and length of lateral roots and root hairs as well as inhibited growth of primary roots. In contrast, the length of the primary root in soybean is also induced by low-P stress. Based on the difference in phenotypic changes in response to low-phosphorus stress, we hypothesized that there is also a difference in methylation changes between soybean and *Arabidopsis*. Therefore, we evaluated genome-wide methylation changes in response to low P using two representative soybean varieties with low-P tolerance and low-P sensitivity. It is noteworthy that only one type of accession was used in previous methylome studies of P starvation response in *Arabidopsis* and rice. Our results revealed that both the low-P-tolerant accession ‘NN’ and the low-P-sensitive accession ‘BG’ exhibit similar dynamic changes in the DNA methylation pattern exposed to low-P availability, which was slightly elevated methylation levels in each methylation sequence context. In contrast, the methylation levels increased by up to 1.5-fold in 7-day-old LP seedlings in comparison with age-matched HP seedlings in *Arabidopsis* [40]. The difference in changes in methylation levels could be a possible explanation for phenotypic differences in the low-P response between *Arabidopsis* and soybean. Furthermore, interestingly, ‘NN’ presented a smaller amount of low-P-induced differentially methylated regions compared with ‘BG’ in each methylation context. The relatively milder methylation changes in ‘NN’ than in ‘BG’ may better maintain genomic stability when suffering low-P conditions. In addition, ‘NN’ and ‘BG’ only shared approximately one-third and one-fifth of the hypermethylated genes and hypomethylated genes, respectively. The more noncommon methylation alterations may contribute to the significantly differential tolerance to low-P stress between ‘NN’ and ‘BG.’

DNA methylation alterations under various stress conditions are often associated with the regulation of gene expression. Accordingly, whether and how DNA methylation is correlated with gene expression under P deficiency is presented and discussed in our study. The results indicate that only a small portion of the low-P stress-induced regions of differential methylation overlapped with genes of differential expression. Most alterations in gene expression were not associated with the corresponding methylation changes. This finding is consistent with the previous observation that a minor fraction of DMRs was correlated with altered gene expression in maize under nutrient deficiencies [65]. This phenomenon could be partially explained by the fact that changes in methylation in some regions do not affect gene expression. It can also be explained that the change of a large number of gene expression is not directly regulated by methylation, and methylation may indirectly affect the transcription network through directly regulating the expression of genes located upstream of the network. This indirect regulation can be regulated by a variety of mechanisms. For example, chilling stress altered the DNA methylation status of *RIPENING INHIBITOR* (*RIN*), *NONRIPENING*, and *COLORLESS NONRIPENING*, which encode transcription factors necessary for ripening, and decreased the transcript levels of these genes and their downstream genes in tomato [66]. Similarly, the methylation and expression levels of the transcription factor gene families *MYB*, *b-ZIP,* and *AP2/DREB* presented significant correlations in soybean during salinity stress [67]. Our results also suggested that many transcription factor genes presented low-P-associated methylation alterations involving members of the *NAC*, *WRKY*, *ERF*, *ARF,* and *bHLH* classes. For instance, a homolog of *auxin response factor 19* (*ARF19*), which positively regulates *PHOSPHATE STARVATION RESPONSE 1*, a central regulatory system of P-responsive genes in *Arabidopsis* roots, was observed to be changed of DNA methylation status and transcripts in our study [68]. It is understandable that plants tend to control TFs compared with regulating structural genes to further regulate biological pathways, which seems to be a more energy-efficient means of coping with environmental stress.

One striking finding of our analysis is that differentially methylated regions are abundantly present in transposable elements, indicating that DNA methylation related to low P availability is necessary for maintaining genome integrity. Furthermore, abundant differentially methylated TEs showed demethylation at each methylation sequence context. In agreement with this finding, it has been recently reported that most TEs were hypomethylated in Zn maize roots [69]. The level of DNA methylation is important for controlling TE activity, and the changed activity of TEs might significantly affect the expression of nearby genes to enhance adaptational processes to abiotic stress, such as P, N, and Zn deficiency [65,70,71]. Accordingly, the dynamic DNA demethylation within TEs observed in this investigation may regulate the transcriptional changes of transposons and proximal genes responding to low-P stress. In addition, the low-P-induced demethylation in TEs, especially in the CHH methylation context, is accompanied by a tremendous reduction in the amount of siRNAs, which was consistent with previous observations of the abundance of siRNAs presented to be positively correlated with DNA methylation in rice and maize subjected to salt stress and Zn deficiency, respectively [69,72]. Furthermore, the methylation levels at each methylation sequence context were significantly higher in siRNA mapping regions than the regions without siRNA mapping. These results indicated that a cross-talk existed between the different methylation pathways, such as the RdDM pathway, to maintain the methylation level.

## 4. Materials and Methods 

### 4.1. Plant Materials and Treatment

The low-P tolerance genotype ‘Nannong 94156′ (NN) and deficient P-sensitive genotype ‘Bogao’ (BG) were grown in hydroponic culture, as described in our previous report [73]. All plants were grown in an artificial climate chamber with a 10 h/14 h (day/night) photoperiod and a temperature cycle of 28 °C/20 °C (day/night). First, the surface-sterilized seeds were germinated in sterile vermiculite. When the two cotyledons were fully expanded, soybean seedlings were transferred into modified 1/2 Hoagland’s nutrient solution for 3 d. Then, half of the seedlings were transferred to modified Hoagland’s nutrient solution at a one-half strength with lacking P (5 µM P, KH_2_PO_4_, low-P, LP), and the other half were transferred into modified Hoagland’s nutrient solution at a one-half strength supplemented with 500 µM P (KH_2_PO_4_, high-P, HP) as controls. These treatments continued for 7 days and the nutrient solution was exchanged every three days. The samples in our study were designated based on the treatments as follows: NN_HP, ‘Nan-nong94-156′ under control conditions; NN_LP, ‘Nannong94-156′ under low-P stress; BG_HP, ‘Bogao’ under control conditions; BG_LP, ‘Bogao’ under low-P stress. Finally, root tissues of nine representative plants from each treatment were harvested and stored at −70 °C for further use.

### 4.2. Bisulfite Sequencing Library Construction

The root tissues of the nine plants of each treatment were pooled to one biological replicate. Genomic DNA was extracted from roots using a modified CTAB method [74]. DNA concentration and integrity were detected by a NanoDrop spectrophotometer and Agarose Gel Electrophoresis, respectively. Briefly, 1 ug of genomic DNA plus unmethylated λ DNA was interrupted into 100–300 bp fragments and purified using a Sonication and MiniElute PCR Purification Kit (QIAGEN, Hilden, Germany). The end-repaired genomic fragments were ligated with a single “A” nucleotide in the 3′ end and then with the methylated sequencing adapters. Then, these fragments were bisulfite converted with the Methylation-Gold Kit (ZYMO, Orange, CA, USA). Finally, the processed DNA fragments were subjected to PCR amplification and double-end sequencing using the Illumina HiSeqTM 4000 platform. 

### 4.3. Read Mapping and Methylation Level Analysis

To obtain high-quality clean reads, raw reads were filtered based on the following rules: (i) removing reads involving more than 10% of unknown nucleotides (N) and (ii) removing low-quality reads containing more than 40% of low-quality (Q-value ≤ 20) bases. The obtained clean reads were mapped to the soybean genome (Glycine max Wm82.a2.v1) using BSMAP software (version: 2.90, Baylor College of Medicine, Houston, TX, USA) by default [75]. Sequencing reads produced during this study have been deposited in the National Center for Biotechnology Information Sequence Read Archive (NCBI SRA accession numbers SRP233333). Then, the methylation level according to the methylated cytosine percentage in the whole genome and in different regions of the genome was calculated using a custom Perl script. Additionally, the methylation profile at flanking 2-kb regions and the gene body (or transposable elements) was plotted according to the average methylation levels of each 100-bp interval to evaluate different methylation patterns in different genomic regions.

### 4.4. Differentially Methylated Regions (DMRs) Analysis

Differentially methylated regions (DMRs) were identified based on the following criteria: (1) at least five methylated cytosines are present in more than one sample; (2) each methylated cytosine is covered by at least four reads; (3) region length is between 40 bp and 10 kb; (4) the distance between adjacent methylated is less than 200 bp; (5) the change of the average methylation level is more than two-fold; (6) Pearson’s chi-square test (χ^2^) value yields a *p*-value ≤ 0.05. To analyze the functional enrichment of genes affected by DMRs, gene ontology (GO) enrichment analysis (http://www.geneontology.org/) and KEGG pathway enrichment analysis (http://www.kegg.jp/kegg/) were conducted for DMR-related genes by the hypergeometric test with a corrected *p*-value ≤ 0.05.

### 4.5. RNA Sequencing and Transcriptome Profiling Analysis

For transcriptome analysis, the root tissues from the nine plants used for methylome analysis were pooled into three samples of three plants each to form three independent biological replicates for each treatment [76]. Total RNA was extracted using Trizol (Life Technologies Inc., Gaithersburg, MD, USA). The RNA quality was tested using an Agilent 2100 Bioanalyzer (Santa Clara, CA, USA), and the quantity was tested using a NanoDrop spectrophotometer (Waltham, MA, USA). The extracted total RNA was purified using Oligo (dT) magnetic beads. Then, the mRNA was fragmented with fragmentation buffer. The short mRNA fragments were reverse transcribed into cDNA by random primers, DNA polymerase I, RNase H, dNTPs, and buffer. Then, the cDNA fragments were purified with a QiaQuick PCR extraction kit. After end-repair, end-addition of A, and ligation to Illumina sequencing adapters, the size of the ligation products was selected by agarose gel electrophoresis followed by PCR amplification and sequencing using the Illumina HiSeqTM 4000 platform. Sequencing raw data was then filtered to remove sequencing adapters and low-quality reads. The clean reads were aligned to the soybean reference genome (Wm82. a2) by TopHat v2.0.3.12 [77]. Sequencing reads generated by this study are available from the NCBI Sequence Read Archive (SRA) Database (accession numbers SRP233239). The calculation of estimated expression abundance was conducted using the Cufflinks package [78]. The FPKM (fragments per kilobase of transcript per million mapped reads) was calculated based on their length and read count, which was used to estimate the transcript abundance of each gene. The edgeR package was applied for differential gene expression analysis (http://www.r-project.org/). The differentially expressed genes were identified according to a fold change ≥2 and a false discovery rate (FDR) <0.05 and were further analyzed by cluster analysis using the “heatmap.2” function in the gplots package of R (https://www.r-project.org/).

### 4.6. Small RNA Sequencing and Data Analysis

Three biological replicates were used for small RNA sequencing. The RNA molecules measuring 18–30 nt were first enriched by polyacrylamide gel electrophoresis (PAGE). The purified small RNA was then ligated to sequencing adapters and performed PCR amplification. The constructed cDNA library was finally sequenced by the Illumina HiSeq TM 4000 platform. After sequencing, the raw data obtained were filtered, including the removal of sequencing adapters and low-quality reads. In addition, reads with identical sequences, including ribosomal RNA, tRNA, small nucleolar RNA, and small nuclear RNA, were removed from the raw data. All of the clean small RNA sequences were mapped to the soybean genome by the SOAP 2.0 program [79]. Only the unique sequences were subjected to subsequent analysis. Sequencing reads generated by this study have been deposited in NCBI’s database of SRA (accession numbers SRP233151).

## 5. Conclusions

Taken together, the results of this work indicate that DNA methylation alterations could affect gene expression in both direct and indirect ways in response to low-P stress in soybean. The indirect alterations in gene expression may be caused by DNA methylation regulating gene expression upstream of the transcription network. Low-P-induced methylation changes were enriched in TEs, and changes in CHH methylation levels in TE regions were accompanied by changes in the amount of siRNA, which indicated that siRNAs could play an important role in regulating TE activity by guiding CHH methylation in TE regions. Our genome-wide perspective revealed unique aspects of methylome changes induced by low P in soybean. These data would be beneficial for studying the epigenetic regulation of abiotic stress responses in plants.

## Figures and Tables

**Table 1:** Summary of genome-wide methylation sequencing data.

Sample	Raw Reads	Clean Reads	Mapped Reads	Mapped Ratio (%)	Sequence Depth	Bisulfite Conversion Efficiency Ratio (%)
NN_HP	444676244	438763364	387477437	88.31%	59.40	99.62%
NN_LP	366193864	360336480	316853049	87.93%	48.57	99.64%
BG_HP	522038932	515810512	458006046	88.79%	70.21	99.65%
BG_LP	404607624	398836454	350842529	87.97%	53.78	99.66%

NN_HP represents ‘Nan-nong94-156′ under control conditions; NN_LP represents ‘Nan-nong94-156′ under low-P conditions; BG_HP represents ‘Bogao’ under control conditions, and BG_LP represents ‘Bogao’ under low-P stress.

## Supplementary Materials

The following are available online at https://www.mdpi.com/1422-0067/21/18/6817/s1.

## Abbreviations

P	Phosphorus
TE	Transposable element
RdDM	RNA-directed DNA methylation
NN	Nannong 94-156
BG	Bogao
LP	Low-P
HP	High-P
DMRs	Differentially methylated regions
GO	Gene Ontology
KEGG	Kyoto Encyclopedia of Genes and Genomes
DMGs	Differentially methylated genes
TFs	Transcription factors
DEGs	Differentially expressed genes
SiRNAs	Small interfering RNAs
