# Supplementary material for: Genome-Wide Analysis Reveals Dynamic Epigenomic Differences in Soybean Response to Low-Phosphorus Stress
Source: Int J Mol Sci. 2020 Sep 17;21(18):6817. doi: 10.3390/ijms21186817 (PMC7555642; doi:10.3390/ijms21186817)
Supplement: Supplementary file 1 [file ijms-21-06817-s001.zip › Supplementary Materials/Supplementary figures.docx]

**
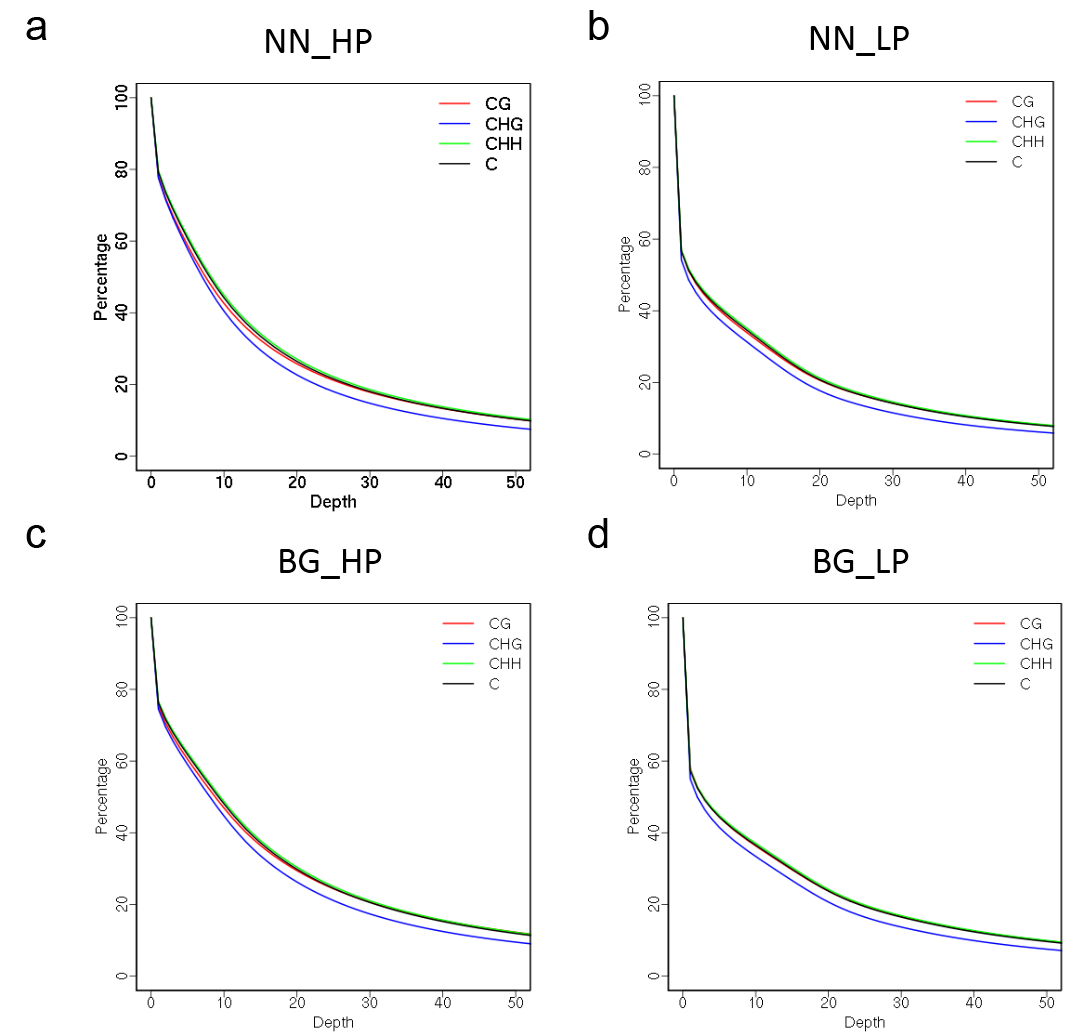
**

**Figure S1.** Sequencing depth and saturation in ‘Nan-nong94-156’ and ‘Bogao’ in response to low-P stress. NN_HP represents ‘Nan-nong94-156’ under control conditions; NN_LP represents ‘Nan-nong94-156’ under low-P conditions; BG_HP represents ‘Bogao’ under control conditions, and BG_LP represents ‘Bogao’ under low-P stress.

**
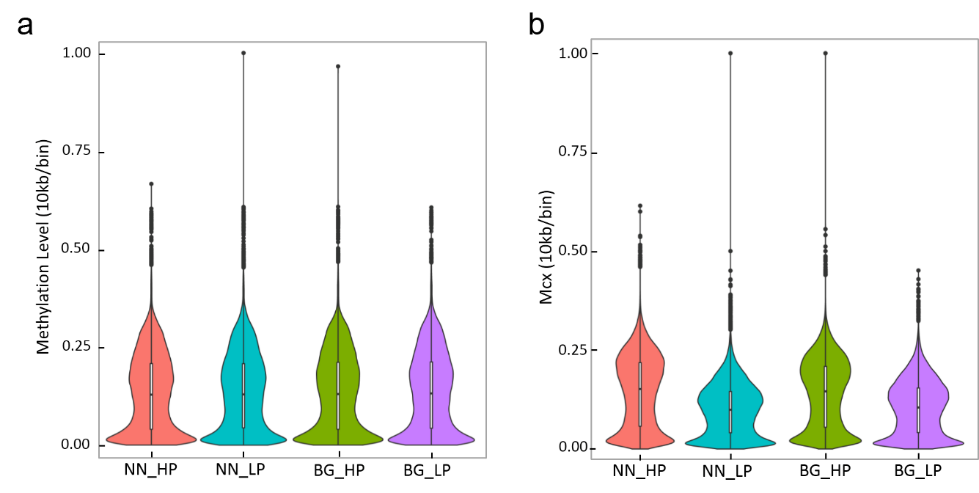
**

**Figure S2.** (**a**) Methylation levels for each of the four samples. The Y-axis of each violin represents methylation levels (10 kb/bin) and the width represents mC abundance at the corresponding methylation level. (**b**) The methylation density of each of the four samples. The Y-axis of each violin represents the percentage of mC density among the total cytosine sites (10 kb/bin). The width of each violin represents the mC abundance at the corresponding methylation density. NN_HP represents ‘Nan-nong94-156’ under control conditions; NN_LP represents ‘Nan-nong94-156’ under low-P conditions; BG_HP represents ‘Bogao’ under control conditions, and BG_LP represents ‘Bogao’ under low-P stress.

**
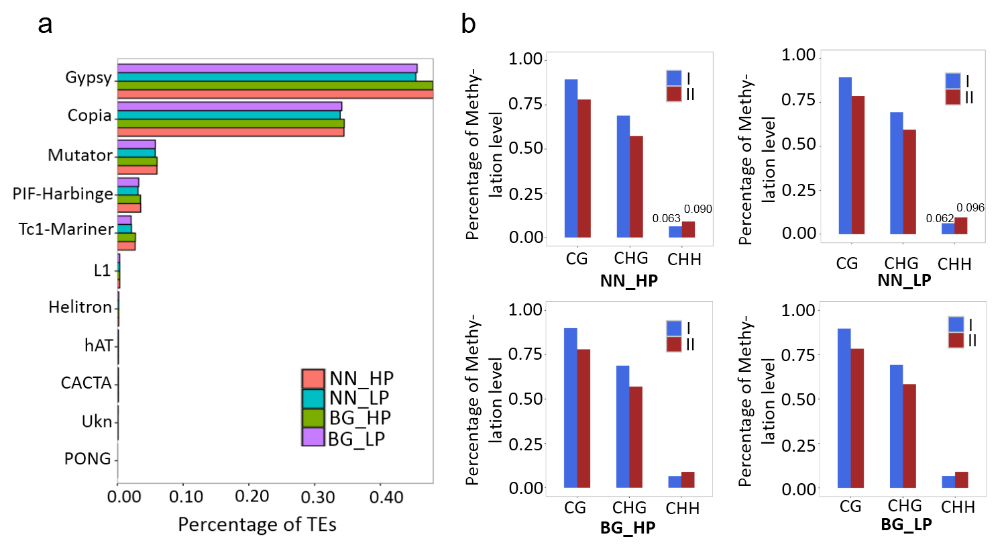
**

**Figure S3.** DNA methylation and TEs.


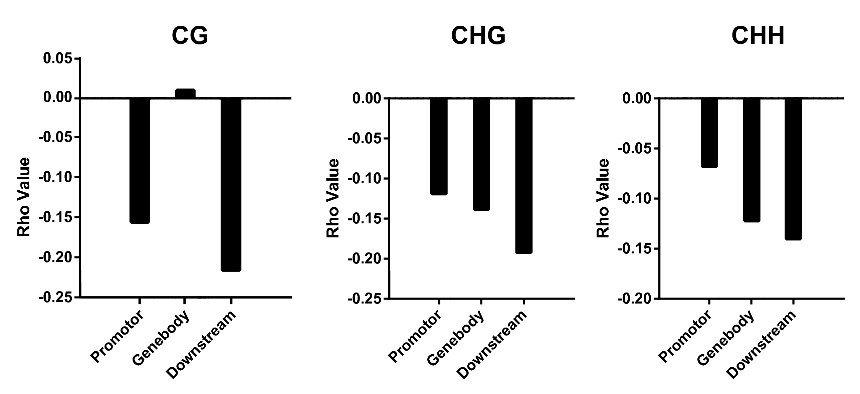


**Figure S4.** Correlation between methylation and gene expression levels in promoter, gene body, and 2kb downstream regions. Rho > 0 means positive correlation, and rho < 0 means negative correlation.

**
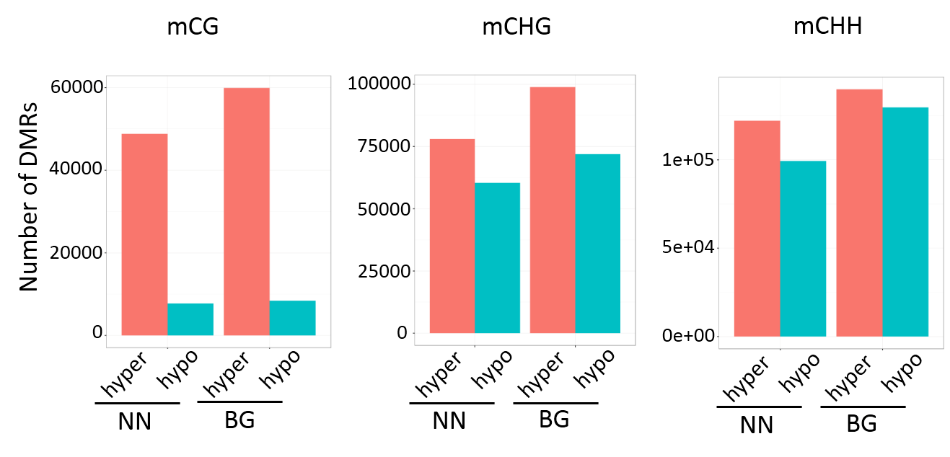
**

**Figure S5.** Number of low-P-induced hyper- and hypo-DMRs in soybean in each methylation context. NN represents ‘Nan-nong94-156’; BG represents ‘Bogao’.

**
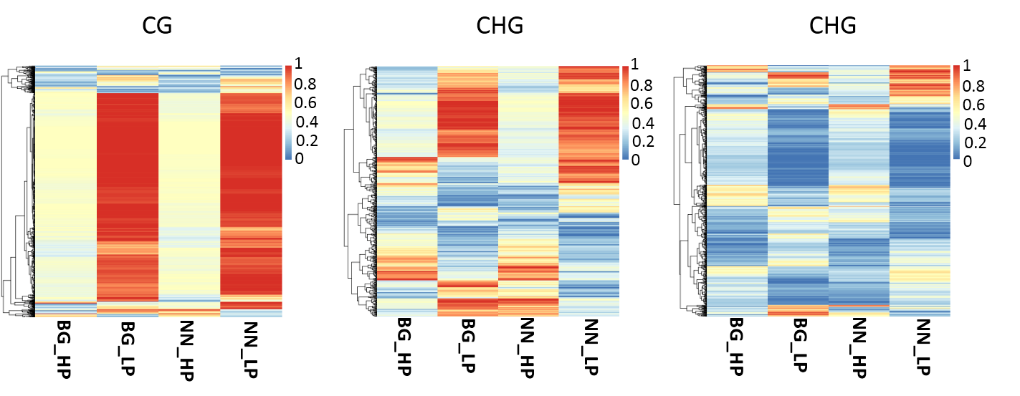
**

**Figure S6.** Heat maps of methylation levels within CG, CHG and CHH DMRs. NN_HP represents ‘Nan-nong94-156’ under control conditions; NN_LP represents ‘Nan-nong94-156’ under low-P conditions; BG_HP represents ‘Bogao’ under control conditions, and BG_LP represents ‘Bogao’ under low-P stress.

**
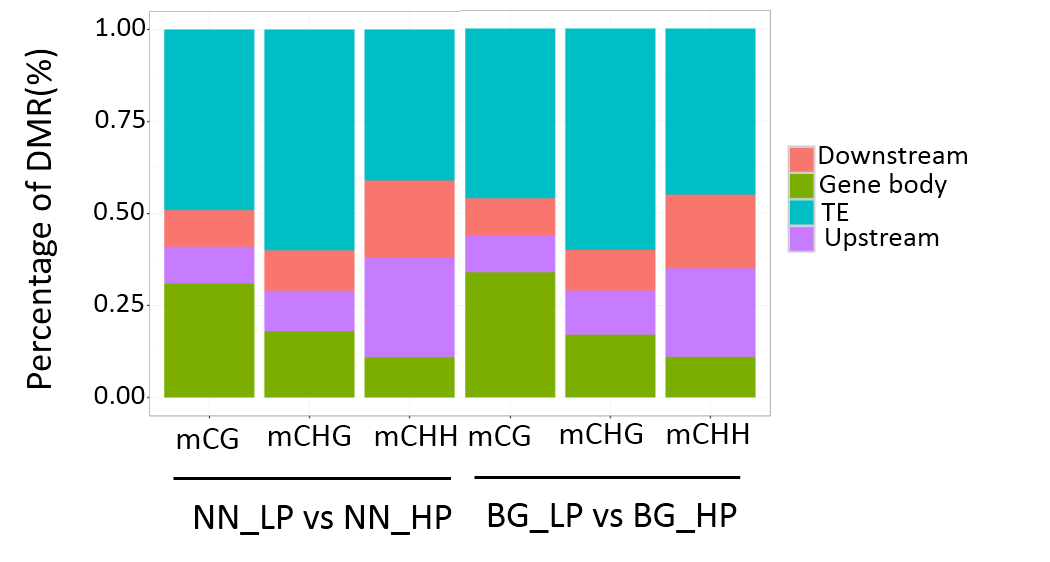
**

**Figure S7.** Percentage of DMR distribution in TE, gene and 2 kb ﬂanking gene regions. NN_LP vs NN_HP, ‘NN’ low-P versus high-P; BG_LP vs BG_HP, ‘BG’ low-P versus high-P.

**
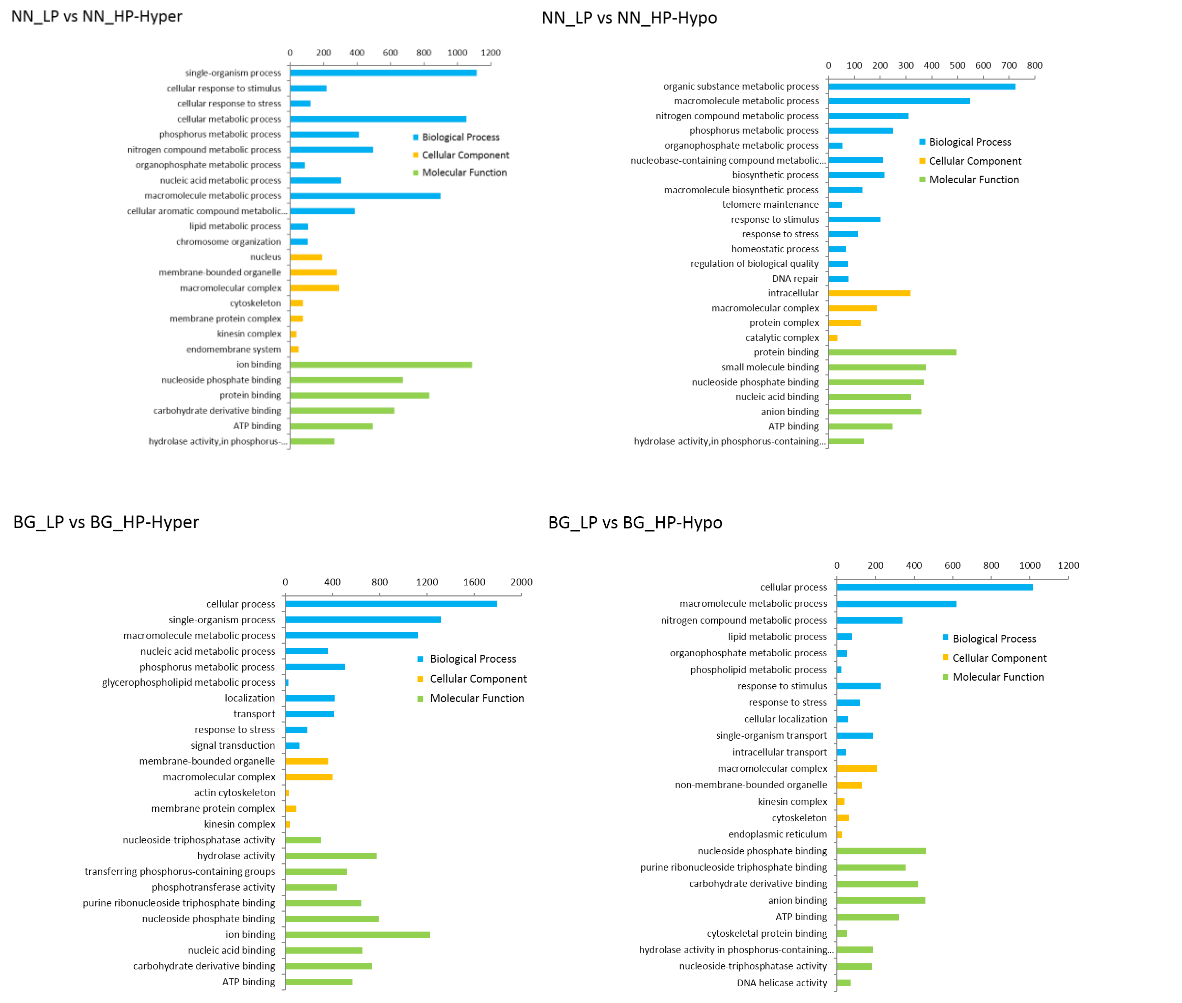
**

**Figure S8.** Gene Ontology (GO) annotation of differentially methylated genes (DMGs) among ‘Nan-nong94-156’ and ‘Bogao’ under low-P stress. NN_LP vs NN_HP, ‘NN’ low-P versus high-P; BG_LP vs BG_HP, ‘BG’ low-P versus high-P.

**
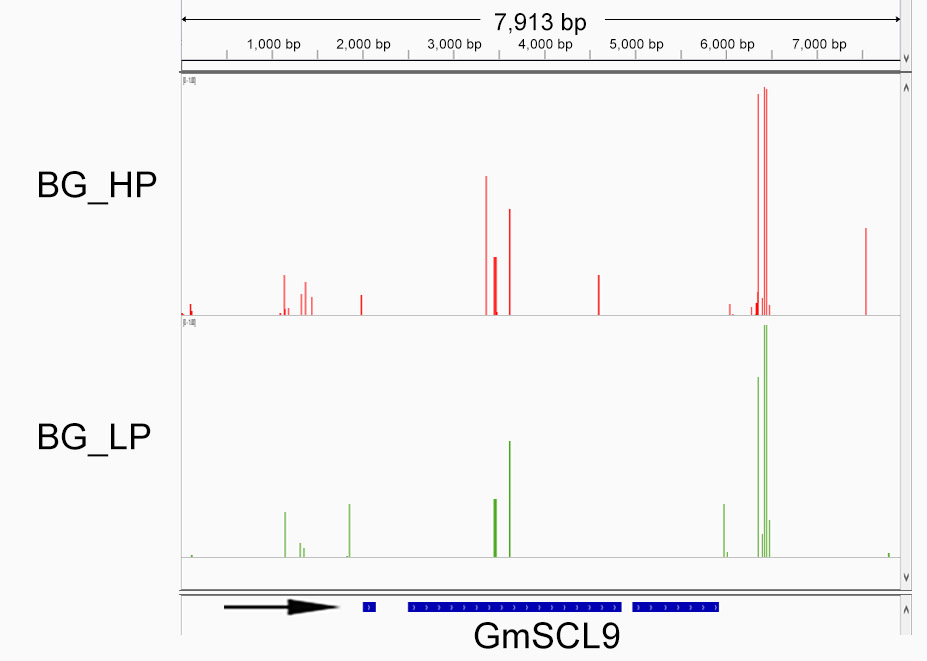
**

**Figure S9.** IGV snapshot of methylation levels in the *GmSCL9* gene body region among BG_LP vs BG_HP (‘BG’ low-P versus high-P).

**
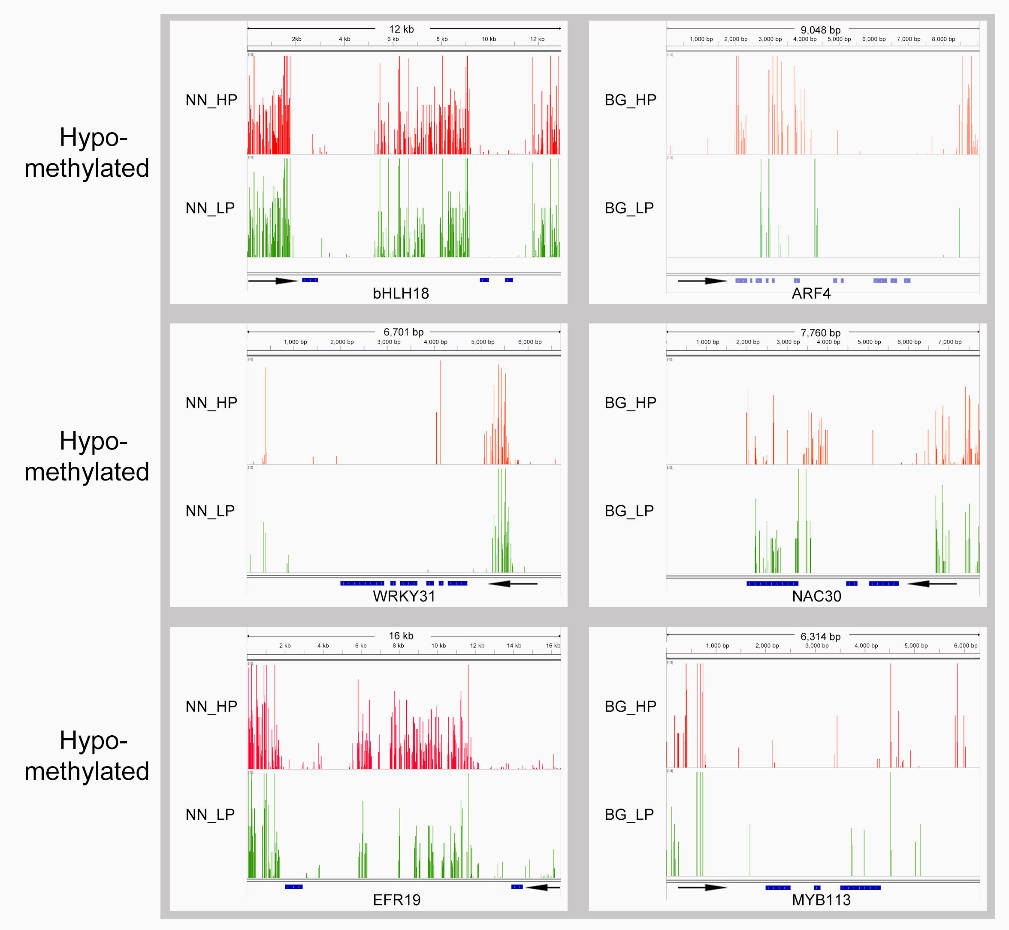
**

**Figure S10.** IGV snapshots of six differentially methylated transcriptional factors (TFs). NN_HP represents ‘Nan-nong94-156’ under control conditions; NN_LP represents ‘Nan-nong94-156’ under low-P conditions; BG_HP represents ‘Bogao’ under control conditions, and BG_LP represents ‘Bogao’ under low-P stress.

**
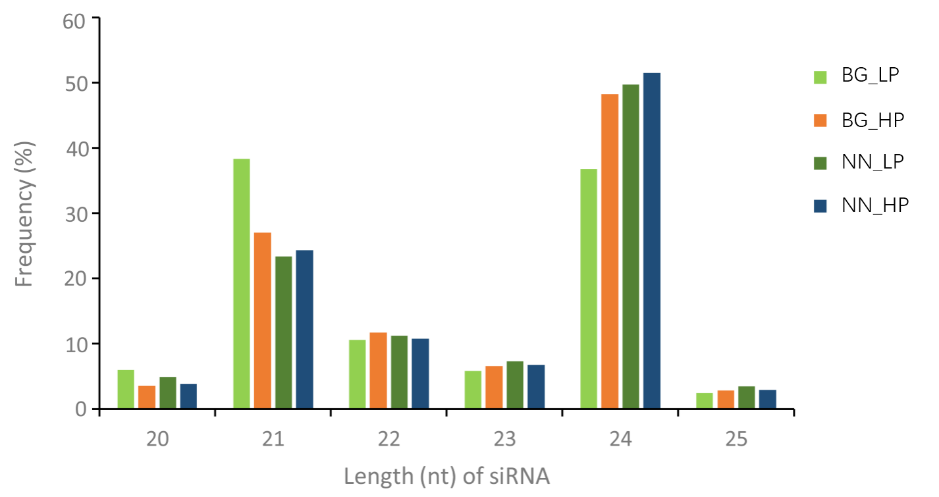
**

**Figure S11.** Size distribution of small RNAs in soybean. NN_HP represents ‘Nan-nong94-156’ at control conditions; NN_LP represents ‘Nan-nong94-156’ under low-P condition; BG_HP represents ‘Bogao’ at control conditions, BG_LP represents ‘Bogao’ under the low-P stress.

**
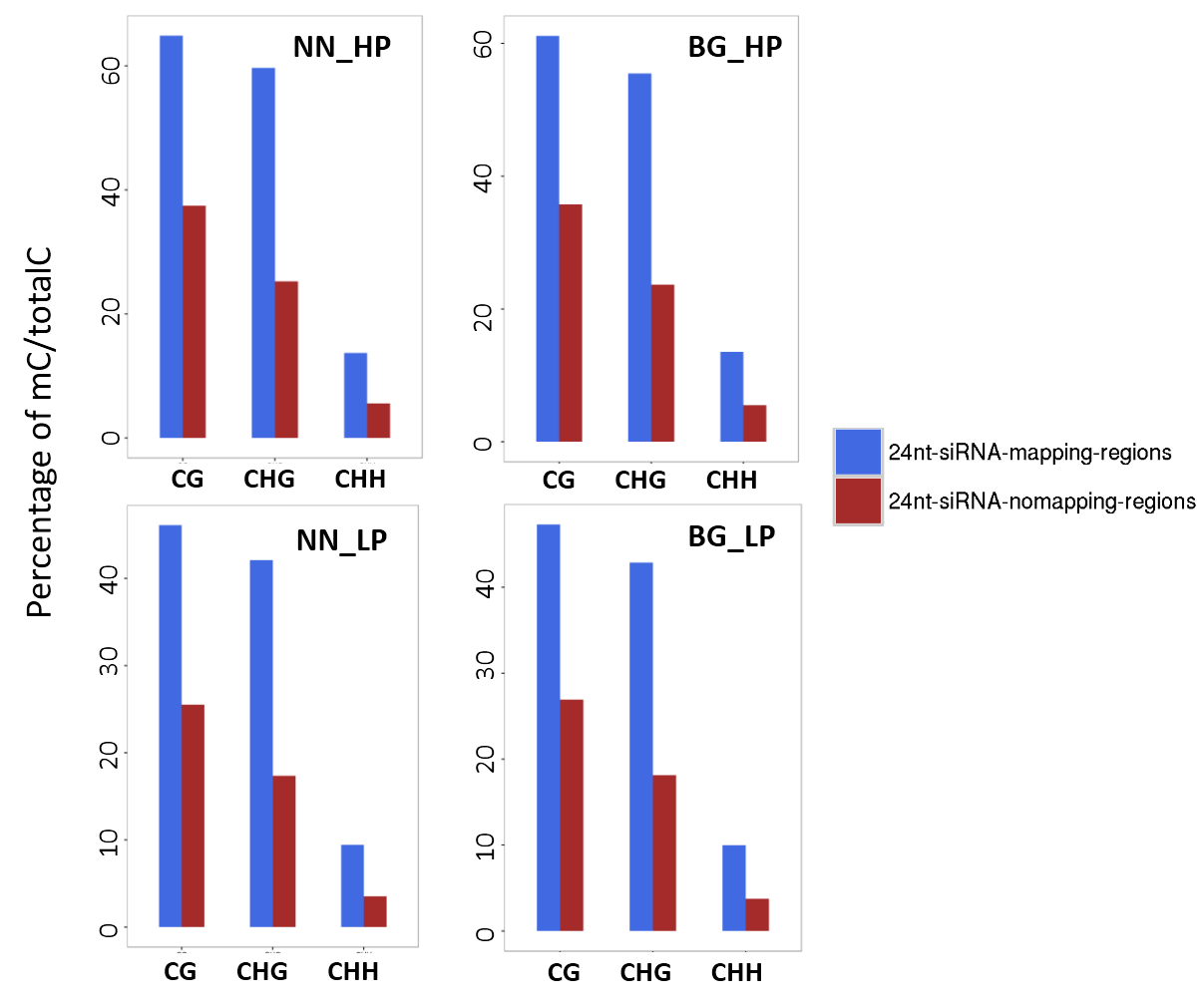
**

**Figure S12.** Comparison of DNA methylation levels between a 24-nucleotide (nt) siRNA uniquely mapping region and without that region in each sequence context in soybean. NN_HP represents ‘Nan-nong94-156’ under control conditions; NN_LP represents ‘Nan-nong94-156’ under low-P conditions; BG_HP represents ‘Bogao’ under control conditions, and BG_LP represents ‘Bogao’ under low-P stress.
